# Assessment of 3D velocity vector fields and turbulent kinetic energy in a realistic aortic phantom using multi-point variable-density velocity encoding

**DOI:** 10.1186/1532-429X-14-S1-W50

**Published:** 2012-02-01

**Authors:** Verena Knobloch, Christian Binter, Utku Gulan, Peter Boesiger, Sebastian Kozerke

**Affiliations:** 1 Institute for Biomedical Engineering, University and ETH Zurich, Zurich, Switzerland; 2 Institute of Environmental Engineering, ETH Zurich, Zurich, Switzerland

## Summary

A multi-point velocity encoding approach for the assessment of velocity vector fields and TKE is shown in this work. The method is applied in an aortic arch phantom under different flow conditions.

## Background

Three-dimensional Phase Contrast (PC) MRI has emerged as a promising non-invasive acquisition technique for assessing velocity vector fields of blood flow [1]. To address the limited sensitivity when velocities are much lower than the encoding velocity v_enc_, three-point acquisition methods with a high v_enc_ and a low v_enc_ acquisition to unwrap the low v_enc_ scan may be employed [2]. However, by using the high v_enc_ data only to control phase unaliasing the approaches are not signal-to-noise ratio (SNR) efficient. This fact becomes relevant in particular when incorporating data undersampling techniques to shorten the long scan times associated with 3D PC-MRI. Accordingly, SNR optimality of encoding and decoding is desired. To this end Bayes’ approaches have been proposed and adapted to PC-MRI [3,4].

In the present work the feasibility of velocity vector field and turbulent kinetic energy (TKE) mapping based on multi-point variable-density velocity encoding with spatiotemporal undersampling is demonstrated on a realistic aortic phantom [5].

## Methods

**Figure 1:**
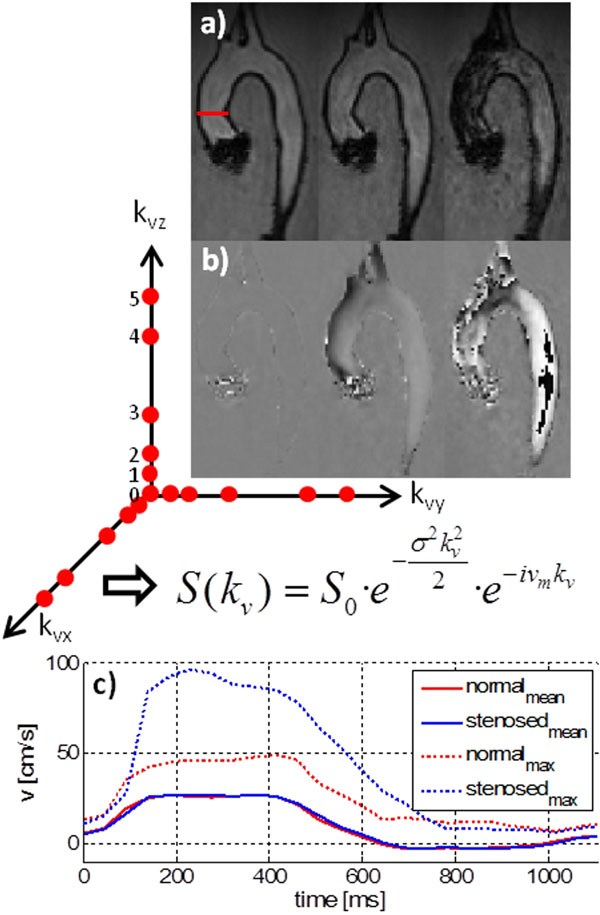
Magnitude (a) and phase (b) images reconstructed from velocity encodes marked in red with 0, 2 and 5 from left to right. The mean velocities v_m_ and TKE are estimated voxelwise. Through-plane velocity profiles measured at the red contour in (a) are shown for the normal and stenosed valve in (c).

An elastic cast of an aortic arch equipped with a mechanical aortic valve (St. Jude Medical Inc., St. Paul, MN, USA) was set up in a pulsatile flow conduit and measured using a velocity encoded, cardiac triggered 3D gradient echo sequence on a 3T Philips Achieva System (Philips Healthcare, Best, The Netherlands). Within a scan time of 33 min, 5 velocity encodings according to v_enc_ = [200, 100, 50, 28, 20] cm/s in each spatial direction plus a non-encoded reference segment were acquired (Fig 1. red dots) with 5x k-t undersampling and 11x6 training profiles with a temporal resolution of 46 ms. Velocities and TKE values [5] were computed using Bayesian parameter estimation [6]. In a second experiment, one leaflet of the valve was fixed in order to simulate a stenotic valve.

## Results

**Figure 2:**
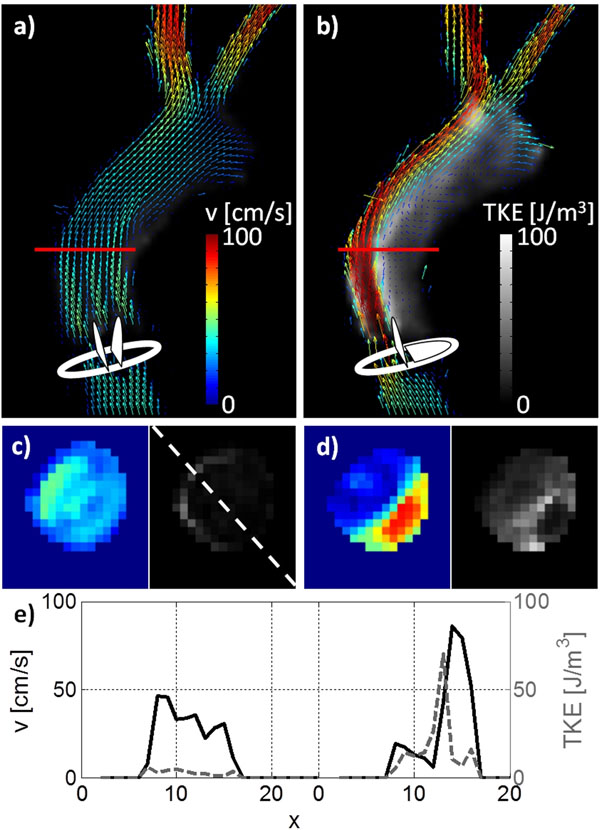
Velocity vector fields and TKE values are shown for t = 322 ms for an axial slice through the normal and stenosed valve (a-b). Absolute velocity and TKE values along profiles marked in (c-d) show an inhomogeneous velocity distribution in the stenosed experiment with high TKE values in the separation plane.

Mean TKE values in the ascending aorta were found to be about 4 times higher for the stenosed valve experiment compared to a normal heart valve. The jet of high velocities up to 100 cm/s is surrounded by increased TKE areas with TKE values > 50 J/m^3^ as it is shown in Fig. 2 b) & d).

## Conclusions

The presented work shows the assessment of velocity vector fields and TKE in a realistic aortic phantom. Using the identical setup comparison of TKE values to data from Particle Tracking Velocimetry (PTV) is possible hence permitting assessment relative to a method of reference for measuring fluctuating velocities at very high temporal resolution.

## Funding

SNF K-32K1_120531/1.
